# Molecular characterization and multilocus genotypes of *Enterocytozoon bieneusi* among horses in southwestern China

**DOI:** 10.1186/s13071-016-1844-3

**Published:** 2016-10-25

**Authors:** Lei Deng, Wei Li, Zhijun Zhong, Chao Gong, Xuehan Liu, Xiangming Huang, Li Xiao, Ruoxuan Zhao, Wuyou Wang, Fan Feng, Yue Zhang, Yanchun Hu, Hualin Fu, Min He, Yue Zhang, Kongju Wu, Guangneng Peng

**Affiliations:** 1 The Key Laboratory of Animal Disease and Human Health of Sichuan Province, College of Veterinary Medicine, Sichuan Agricultural University, Chengdu, Sichuan Province 611130 China; 2 Chengdu Giant Panda Breeding Research Base, Chengdu, Sichuan Province 625001 China

**Keywords:** *Enterocytozoon bieneusi*, ITS gene, MLST, Horse, Zoonotic

## Abstract

**Background:**

*Enterocytozoon bieneusi* is one of the most prevalent causative species of diarrhea and enteric diseases in various hosts. *E. bieneusi* has been identified in humans, mammals, birds, rodents and reptiles in China, but few studies have reported *E. bieneusi* in horses. Therefore, the present study was conducted to assess the prevalence, molecular characteristics and zoonotic potential of *E. bieneusi* among horses in southwestern China.

**Findings:**

Three hundred and thirty-three fecal specimens were collected from horses on five farms in the Sichuan and Yunnan provinces of southwestern China. The prevalence of *E. bieneusi* was 22.5 % (75/333), as determined by nested polymerase chain reaction and sequencing analysis of the internal transcribed spacer region of the ribosomal RNA gene of *E. bieneusi*. Altogether, 10 genotypes were identified among the 75 *E. bieneusi*-positive samples: four of these genotypes were known (horse1, horse2, SC02 and D) and six were novel (SCH1-4 and YNH1-2). Multilocus sequence typing using three microsatellites (MS1, MS3 and MS7) and one minisatellite (MS4) revealed three, two, three and three genotypes at these four loci, respectively. In phylogenetic analysis, all the genotypes of *E. bieneusi* obtained in this study were clustered into three distinct groups: D, SC02 and SCH1-3 were clustered into group 1 (zoonotic potential); SCH4 was clustered into group 2 (cattle-hosted); whereas horse2, YNH1 and YNH2 were clustered into group 6 (unclear zoonotic potential).

**Conclusions:**

This is the first report of *E. bieneusi* among horses in southwestern China. This is also the first multilocus genotyping analysis using microsatellite and minisatellite markers of *E. bieneusi* in horses. The presence of genotype D, which was previously identified in humans, and genotypes SC02 and SCH1-3, which belong to potential zoonotic group 1, these results indicate that horses are a potential source of human *E. bieneusi* infections in China.

## Background

Microsporidia are parasitic fungi that cause gastroenteritis in invertebrate and vertebrate taxa [1]. The phylum Microsporidia contains approximately 1300 species in 160 genera, and at least 17 species within nine genera have been identified in humans [2]. *Enterocytozoon bieneusi* is the most common microsporidian species, and is responsible for more than 90 % of reported cases of human microsporidiosis [3]. *Enterocytozoon bieneusi* was first reported in enterocytes of a Haitian patient with AIDS [4]. In humans, *E. bieneusi* can cause chronic life-threatening diarrhea and wasting in immunocompromised individuals, whereas it seems to cause self-limiting diarrhea and malabsorption in healthy individuals [5]. In addition, *E. bieneusi* has been reported in various wild, domestic and companion animals, as well as in birds worldwide [6, 7].

Sequence analysis of the internal transcribed spacer (ITS) region of the rRNA gene has been generally regarded as a standard method for the genotyping of *E. bieneusi* isolates in humans and animals [3, 8]. To date, more than 240 genotypes have been identified in various animal hosts [6, 9]. By phylogenetic analysis, the ITS genotypes of *E. bieneusi* have been divided into nine different groups [10, 11]. A large cluster (group 1) includes 94 % of the published genotypes of *E. bieneusi*, and has been established to have zoonotic potential [12]. In contrast, the remaining eight major clusters (groups 2–9) have mostly been found in specific hosts and wastewater [13, 14]. To better understand the taxonomy and molecular characteristics of *E. bieneusi*, high-resolution multilocus sequence typing (MLST) using three microsatellites (MS1, MS3 and MS7) and one minisatellite (MS4) as markers was developed [15].

In China, horses are used frequently for work and social activities. Horses commonly live in close consociation with humans and their environmental shedding of *E. bieneusi* spores may be a threat to public health. However, only one previous study has reported *E. bieneusi* infection in grazing horses in the Xinjiang Uyghur Autonomous Region [16]. The aims of the present study were to investigate the prevalence and molecular characteristics of *E. bieneusi* from horses in the Sichuan and Yunnan provinces of China, and evaluate the potential role of horses in the transmission of human microsporidiosis.

## Methods

### Collection of fecal specimens

During the period from August 2015 to April 2016, a total of 333 fecal samples were collected from horses on five farms located in the Sichuan (3 farms, 156 horses) and Yunnan (2 farms, 177 horses) provinces of southwestern China (Table 1). Farms were selected only based on the owners’ willingness to participate and the accessibility of animals for sampling. Each sample was collected from each horse immediately after they defecated onto the ground, using a sterile disposal latex glove, and then was placed into individual 50 ml plastic containers. The ages of the animals ranged from 3 months to 23 years, and none of them had any apparent clinical signs of illness at the time of sampling.

**Table 1:** Prevalence and distribution of *E. bieneusi* genotypes by geography in southwestern China

Location (province)	Farm	No. examined	No. positive (%)	Genotype (*n*)
Chengdu (Sichuan)	Farm 1	48	5 (10.4)	SC02 (3); SCH1 (1); SCH4 (1)
	Farm 2	52	5 (9.6)	horse1 (5)
	Farm 3	56	15 (26.8)	SC02 (13); SCH2 (1); SCH3 (1)
	Subtotal	156	25 (16.0)	SC02 (16); horse1 (5); SCH1 (1); SCH2 (1); SCH3 (1); SCH4 (1)
Kunming (Yunnan)	Farm 4	12	2 (16.7)	horse2 (2)
	Farm 5	165	48 (29.1)	horse2 (37); horse1 (7); SC02 (1); D (1); YNH1 (1); YNH2 (1)
	Subtotal	177	50 (28.2)	horse2 (39); horse1 (7); SC02 (1); D (1); YNH1 (1); YNH2 (1)
Total		333	75 (22.5)	horse2 (39); SC02 (16); horse1 (13); D (1); SCH1 (1); SCH2 (1); SCH3 (1); SCH4 (1); YNH1 (1); YNH2 (1)

### DNA extraction

Prior to DNA extraction, the fecal specimens were washed three times with distilled water. Genomic DNA was extracted from approximately 200 mg of washed fecal specimens, using an EZNA® Stool DNA kit (Omega Biotek, Norcross, GA, USA) according to the manufacturer’s recommended protocol. DNA was eluted in 200 μl of absolute ethanol and stored at -20 °C until used for PCR analysis.

### PCR amplification

*Enterocytozoon bieneusi* was detected by nested PCR amplification of a 389 bp nucleotide fragment of the rRNA gene. The PCR amplification primers and amplification conditions of the ITS gene were previously described by Sulaiman et al. [17]. Positive specimens were further characterized by MLST analyses, using MS1, MS3, MS4 and MS7, according to the methods described by Feng et al. [15]. TaKaRa Taq™ DNA Polymerase (TaKaRa Bio, Otsu, Japan) was used for all PCR amplifications. A negative control with no DNA added was included in all PCR tests. All secondary PCR products were subjected to electrophoresis on a 1 % agarose gel containing ethidium bromide.

### Nucleotide sequencing and analysis

The secondary PCR products of the anticipated size were directly sequenced by Life Technologies (Guangzhou, China), using a BigDye® Terminator v3.1 cycle sequencing kit (Applied Biosystems, Carlsbad, CA, USA). Sequence accuracy was confirmed by two-directional sequencing and the sequencing of a new PCR product if necessary.

The obtained sequences were aligned with reference sequences downloaded from GenBank using the Basic Local Alignment Search Tool (BLAST) (http://www.ncbi.nlm.nih.gov/BLAST/) and ClustalX 1.83 (http://www.clustal.org/) to determine the genotypes of *E. bieneusi*. The genotypes that were identified as being identical to those downloaded from GenBank were assigned with their previously published names. Obtained genotypes with single nucleotide substitutions, deletions or insertions compared with the previously reported genotypes were considered novel and named according to the established nomenclature system [8].

### Phylogenetic analysis

A neighbor-joining tree was constructed to assess the genetic relationships among the *E. bieneusi* genotypes obtained in the present study and those identified in previous studies, using the software Mega 6 (http://www.megasoftware.net/), and the evolutionary distances were calculated using the Kimura two-parameter model. The reliability of these trees was assessed by bootstrap analysis with 1000 replicates.

### Statistical analysis

The Chi-square test was performed to compare the *E. bieneusi* infection rates, and differences were considered significant when *P* < 0.05.

## Results and discussion

In the present study, 75 (22.5 %) out of the 333 horses were identified as *E. bieneusi*-positive. Horses at every tested farm showed evidence of *E. bieneusi* prevalence (Table 1), with the highest prevalence in Farm 5 (29.1 %), which serves as a supplier of horses to other farms; the horses at Farm 5 spent most of their time on the pasture. The second highest prevalence was at Farm 3 (26.8 %), where horses are widely used for transportation. The horses at Farm 4 (16.7 %) are mainly used in experimental research, such as that on *Clostridium tetani*. Farm 1 (10.4 %) and Farm 2 (9.6 %) both act as equestrian clubs, and the horses are largely used for horseback riding, horse racing and show jumping. The differences in the prevalence of *E. bieneusi* among horses from different farms may be explained by different farm management systems. The prevalence in horses > 3 years of age was higher (25.0 %) than that in horses < 1 year of age (17.1 %) (Table 2), but the difference was not significant (*χ* ^2^ = 1.193, *df* = 2, *P* > 0.05). This result is consistent with previous findings in horses [16, 18, 19]. A non-significant difference in infection rates was observed between males (18.5 %) and females (25.7 %) (*χ* ^2^ = 2.419, *df* = 1, *P* > 0.05) (Table 2), which is consistent with recent studies [16, 19].

**Table 2:** Prevalence and distribution of *E. bieneusi* genotypes by age and sex

Group	No. examined	No. positive (%)	*E. bieneusi* genotype (*n*)
Age (years)			
< 1	35	6 (17.1)	horse2 (2); SC02 (3); horse1 (1)
2–3	154	33 (21.4)	horse2 (23); horse1 (4); D (1); SC02 (1); YNH1 (1); YNH2 (1); SCH1 (1); SCH3 (1)
> 3	144	36 (25.0)	horse2 (14); horse1 (7); SC02 (13); SCH2 (1); SCH4 (1)
Sex			
Female	187	48 (25.7)	horse2 (29); horse1 (6); SC02 (9); D (1); SCH1 (1); SCH2 (1); SCH4 (1)
Male	146	27 (18.5)	horse2 (10); horse1 (6); SC02 (8); YNH1 (1); YNH2 (1); SCH3 (1)

Sequence analysis of the ITS region of *E. bieneusi* isolates showed 10 genotypes among 75 *E. bieneusi*-positive specimens, including four known (horse1, horse2, SC02 and D) and six novel (which we have named SCH1-4 and YNH1-2) genotypes. Genotypes horse1 and horse2 have frequently been described in horses and have been identified in Colombia [20], the Czech Republic [18], Algeria [19] and China [16]. Genotype D is the most common genotype in humans and animals and has been detected in over 25 animal species [12, 21–24]. Genotype SC02 was the first to be identified in horse and has been found to have various hosts, such as Tibetan blue bear, Asiatic black bear, sun bear and northern raccoon [12].

**Fig. 1:**
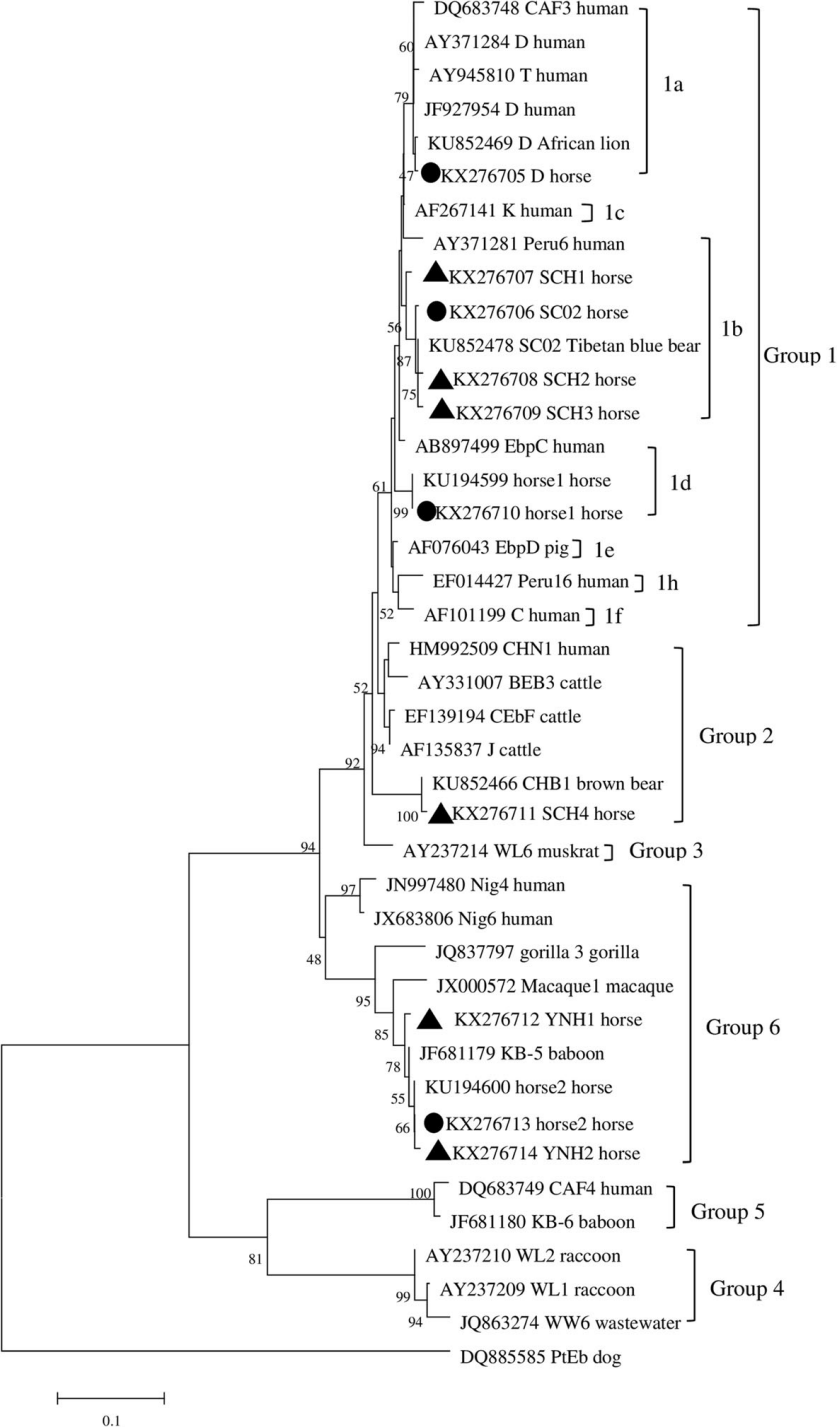
Phylogenetic relationships of *Enterocytozoon bieneusi* groups. The relationships between *E. bieneusi* genotypes identified in this study and other known genotypes deposited in the GenBank were inferred by a neighbor-joining analysis of internal transcribed spacer sequences based on genetic distance by the Kimura-2-parameter model. The numbers on the branches represent percent bootstrapping values from 1000 replicates, with values of more than 50 % shown in the tree. Each sequence is identified by its accession number, genotype designation and host origin. Genotypes marked with *black triangles* and *black circles* are novel and known genotypes identified in this study, respectively

A phylogenetic analysis based on ITS gene sequences showed the genetic diversity of the obtained genotypes of *E. bieneusi* and their relationships with the known genotypes (Fig. 1). The six genotypes (horse1, D, SC02 and SCH1-3) belonged to group 1, indicating their potential for zoonotic transmission. The novel genotype SCH4 was clustered into group 2, which consists almost entirely of genotypes from cattle, but some genotypes (I and BEB6) were also detected in humans [25, 26]. The remaining three genotypes (horse2, YNH1 and YNH2) were clustered into group 6, which was first reported in urban wastewater [14]. The genotypes gorilla 3 in gorillas and Nig4 and Nig6 in humans were also clustered into group 6 [26, 27]. Thus, it is difficult to assess the potential for the zoonotic transmission of the novel genotypes in groups 2 and 6.

Recently, a high-resolution MLST tool has been developed to further improve taxonomy and population genetics of *E. bieneusi* [15]. A high multilocus genotype (MLG) diversity was observed in the same ITS region in previous studies [12, 21]. In the present study, of the 75 specimens positive for ITS, 13, 5, 14 and 9 were successfully amplified at MS1, MS3, MS4 and MS7, respectively, but only five samples were simultaneously positive at all four loci. Sequence analysis identified three, two, three and three novel genotypes at the MS1, MS3, MS4 and MS7 loci, respectively. Analysis of the five samples that were positive at all four gene loci formed three distinct MLGs, namely MLG1-3 (Table 3). These findings showed the genetic diversity of *E. bieneusi* in horse.

**Table 3:** Multilocus sequence typing of *E. bieneusi* isolates from horses in southwestern China

Location	Farm	ITS genotype	Multilocus genotype				
			MS1	MS3	MS4	MS7	MLGs
Kunming	Farm 5	horse2	Type I	Type I	Type I	Type I	MLG1
	Farm 5	horse2	Type I	Type I	Type I	Type I	MLG1
	Farm 5	horse1	Type II	Type I	Type II	Type II	MLG2
	Farm 5	horse2	Type I	Type I	Type I	Type I	MLG1
Chengdu	Farm 5	horse1	Type III	Type II	Type I	Type III	MLG3

## Conclusions

The data obtained in the present study indicates that *E. bieneusi* infection is prevalent among horses in southwestern China. The observation of six genotypes (horse1, D, SC02 and SCH1-3) clustered into group 1 suggests that horses may serve as reservoir hosts for the zoonotic transmission of *E. bieneusi*. The genetic diversity of *E. bieneusi* was observed by MLST analysis in horses for the first time, and three distinct MLGs were found. Since the specific routes of transmission of *E. bieneusi* remain unknown and there are no effective drugs for the complete treatment of *E. bieneusi* infection in humans or animals, farm managers should be advised to take measures to control environmental contamination.

## Abbreviations

ITS: Internal transcribed spacer
MLGs: Multilocus genotyping
MLST: Multilocus sequence typing
